# The distribution of phthalate esters in indoor dust of Palermo (Italy)

**DOI:** 10.1007/s10653-013-9544-9

**Published:** 2013-06-27

**Authors:** Santino Orecchio, Roberta Indelicato, Salvatore Barreca

**Affiliations:** 1 Dipartimento di Scienze e Tecnologie Biologiche, Chimiche, Farmaceutiche, Università di Palermo, Parco Orleans II, Ed. 17, 16-90128 Palermo, Italy; 2 Dipartimento di Fisica e di Chimica, Università di Palermo, Parco Orleans II, Ed. 17, 16, 90128 Palermo, Italy

**Keywords:** Indoor dust, Phthalates esters, GC–MS, Contaminants, Italy

## Abstract

In this work, phthalic acid esters (PAEs): dimethyl phthalate (DMP), diethyl phthalate (DEP), di-*n*-butyl phthalate, benzyl butyl phthalate, bis(2-ethylhexyl) phthalate, and di-*n*-octyl phthalate in indoor dust (used as passive sampler) were investigated. The settled dust samples were collected from thirteen indoor environments from Palermo city. A fast and simple method using Soxhlet and GC–MS analysis has been optimized to identify and quantify the phthalates. Total phthalates concentrations in indoor dusts ranged from 269 to 4,831 mg/kg d.w. (d.w. = dry weight). The data show a linear correlation between total PAEs concentration and a single compound content, with the exclusion of the two most volatile components (DMP and DEP) that are present in appreciable amounts only in two samples. These results suggest that most of the PAEs identified in the samples of settled dust originate from the same type of material. This evidence indicates that, in a specific indoor environment, generally is not present only one compound but a mixture having over time comparable percentages of PAEs. Consequently, for routine analyses of a specific indoor environment, only a smaller number of compounds could be determined to value the contamination of that environment. We also note differences in phthalate concentrations between buildings from different construction periods; the total concentration of PAEs was higher in ancient homes compared to those constructed later. This is due to a trend to reduce or remove certain hazardous compounds from building materials and consumer goods. A linear correlation between total PAEs concentration and age of the building was observed (*R* = 0.71).

## Introduction

Scientists have hypothesized indoor dust that may be closely linked with health effects, especially in children and adults with chronic lung disease through time (Adgate et al. 2003), because people spend more than 80 % of their time in indoor environments (Benner et al. 1989). In particular, house dust is a repository of many kinds of pollutants (Raiyani et al. 1993; Cizdziel and Hodge 2000; Becker et al. 2004; Maerteens et al. 2004; Mannino and Orecchio 2008), and it is necessary to evaluate indoor contaminants concentrations and distributions in order to assess total human exposure to them.

Among the hazardous pollutants, phthalate esters, also called phthalic acid esters (PAEs), are important owing to their carcinogenicity and reproductive effects (Kavlock et al. 2002). Phthalates, generally, are colorless and odorless liquids having high boiling points (228–380 °C), low volatility, and showing insolubility in water and predominantly fat solubility. With the exception of dimethyl phthalate, which belong to the group of VOCs (volatile organic compounds), PAEs are classified as semi-volatile organic compounds. Phthalates are introduced into the environment only by anthropogenic sources (Wormuth et al. 2006). PAEs are emitted into the atmosphere as particulates and gases (Weschler et al. 2008).

Limited data exist about the gas-particle partitioning of these chemicals in urban environment, almost certainly owing to difficulties in analysis of PAEs in atmosphere (Wang et al. 2008a). Some authors (Weschler et al. 2008) argue that the less volatile PAEs are more likely to be deposited on the indoor surfaces bound to particles in wet and dry deposition. For compounds of intermediate vapor pressure, a temperature-dependent gas/particle portioning of PAEs will occur, and thus, they are subject to both wet and dry deposition in gaseous and particle-bound form. The transport, residence time, fate, and reactions of PAEs in atmosphere are widely controlled by their gas-particle partitioning (Cousins and Mackay 2001). The partitioning of semi-volatile organic compounds on aerosols, consisting of a significant liquid-like layer, has been studied using several models showed that for absorptive gas/particle partitioning, the equilibrium-partitioning coefficient K_p_ depends on many factors (particle and gas-phase concentrations of the compound, total suspended particulate matter concentration, temperature, etc.) (Chandramouli et al. 2003).

Indoor environments increase the lifetime of pollutants adsorbed to the dust by minimizing or eliminating the natural decomposition processes catalyzed by natural light and rain (Cizdziel and Hodge 2000). Compounds with higher molecular weights, such as bis(2-ethylhexyl) phthalate (DEHP), are largely used as additives and plasticizers, while those with lower molecular weights (diethyl, di-*n*-butyl and dimethyl phthalate) are components of industrial solvents, adhesive, wax, ink, pharmaceutical products, insecticide materials, and cosmetic (Schettler 2006; Koniecki et al. 2011). DEHP was found in medical disposals devices and in a number of medicine coatings. Some compounds are contained in cleaning solutions for contact lenses (Pérez-Feás et al. 2001) and in food-packaging films (Bonini et al. 2008).

Phthalates are not chemically but only physically bound to the polymer chains; hence, they may be leached into the environment and are ubiquitously found in air, water, soils, and sediments (Yuan et al. 2002; Sha et al. 2007; Wang et al. 2008a, b; Zeng et al. 2008, 2009; Xia et al. 2011). People exposure to PAEs may arise from toys and child-care articles, building materials and home furnishing, car interiors, clothing and via medical devices, and food-contact materials (Clausen et al. 2003, 2004).

A relationship between phthalate concentrations in dust collected from the children’s bedrooms and asthma and allergies in children has been previously reported (Patriarca et al. 2000; Bornehag et al. 2004; Kolarik et al. 2008a). In particular, a case–control study was carried out by some authors (Bornehag et al. 2004) within a group of 10,852 children. Within this group, the researchers selected 198 cases with persistent allergic symptoms and 202 controls without allergic symptoms. A clinical and a technical team investigated each child and her or his environment. The researchers founded higher median concentrations of BBzP in dust among cases than among controls. Analyzing statistically the case group by symptoms showed that the presence of BBzP was associated with rhinitis and eczema, whereas DEHP was associated with asthma.

Inhalation of dust can occur when it is suspended by activities such as cleaning, playing, or walking through a room. Phthalates levels in house dust are generally higher than in yard and foundation soil (Cizdziel and Hodge 2000). This is because indoor dust can potentially remain undisturbed for several years, and natural decomposition processed and catalyzed by natural light and rain are minimized or eliminated in indoor environments (Cizdziel and Hodge 2000).

There are many investigations performed to assess mass levels and chemical characteristics of indoor particulates and their relationships with the corresponding outdoor environments (Guidotti et al. 1998; Jones et al. 2000; Teil et al. 2006). They clearly demonstrated that the correlations of the indoor and outdoor particles by count or mass concentrations varied widely. Different areas with different particle source emissions and meteorological characteristics as well as infiltration of particles into indoors are critical. However, there are few investigators engaged in evaluating the distributions of phthalate esters in indoor dust (Clausen et al. 2003; Kolarik et al. 2008a, b; Abb et al. 2009), and very limited data, known to us, are available on PAEs in Italian household dust. Several studies have reported high exposure levels to contaminants indoors, particularly in poorly ventilated houses (Butte and Heinzow 2002; Mannino and Orecchio 2008).

Normally, common pollutants (NO_x_, SO_2_, CO, O_3_, etc.) in indoor air are analyzed using real-time monitoring instruments that sample and analyze it, but is not available any real-time monitoring instrument for PAEs. A practical problem in analyzing environmental contaminants is their very low concentration near or below the detectable analytical limits, at which they often occur. In air, concentrations vary widely over time. Interpreting trace contaminants concentrations in air and predicting the threat they pose to human life under variable physical–chemical conditions are very difficult.

The purpose of this work is to present a simple method to analyze phthalic esters in indoor-settled dust, used as a passive sampler, and to investigate on PAEs concentrations in several indoor environments. There are many advantages of using settled indoor dusts as passive accumulators (Mannino and Orecchio 2008). They essentially provide information on the average variation in time and space of the concentrations of contaminants in the considered area.

The compounds analyzed in this paper are the most used in industrial processes which are expected to be present in indoor environments: dimethyl phthalate (DMP), diethyl phthalate (DEP), di-*n*-butyl phthalate (DnBP), benzyl butyl phthalate (BBzP), bis(2-ethylhexyl) phthalate (DEHP), and di-*n*-octyl phthalate (DnOP). Other compounds (PAHs, PCBs, metals, etc.) (Culotta et al. 2002, 2005, 2007; Gianguzza et al. 2006a, 2008; Gianguzza and Orecchio 2006b; Orecchio 2007, 2010; Orecchio et al. 2013) which are present in the environmental samples (dusts, particulates, natural waters, sediments, etc.) often complicate the analysis of phthalate esters in environmental matrices. To avoid these problems, the GC–MS method in single-ion monitoring (SIM) mode was used.

We present the results relative to indoor-settled dust collected from homes of Palermo in order to evaluate the magnitude and distribution of concentrations inside common environments and suggest the possible origins of the considered contaminants. This investigation characterizes human real exposure to phthalate esters indoor stations. Our study has been carried out in the area of Palermo, which is a tourist and commercial town with a population of about 800,000 inhabitants.

## Materials and methods

### Sample collection

**Fig. 1:**
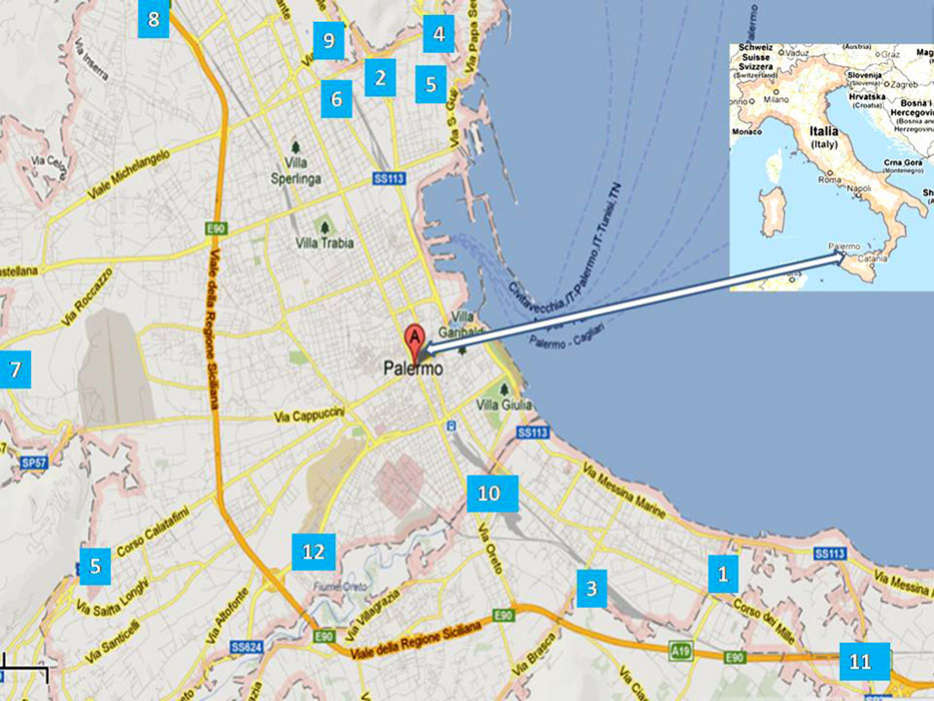
Sampling stations

Palermo is a coastal city located in Southern Italy (Fig. 1). The weather is warm (annual mean temperature is 23 °C), and relative humidity is high (annual mean = 71.5 %).The ventilation of the indoor environments is generally provided by windows and in few cases by air-conditioning systems.

In three cases, PAEs were measured in the atmospheric dust to verify if the outdoor pollutants could have influence on the indoor concentration of phthalates. Particulate samples were collected on February 2013 at sampling stations no. 12 (Fig. 1) and characterized by different anthropic activities. Sampling was performed according to European Standard EN12341. Samples were collected using a sampler (Explorer or mod. ZB1, Zambelli, Italy), a sampling inlet head (Zambelli), operating at a constant sampling rate of 38.3 L/m (2.3 m^3^/h). The sampling time was 24 h. Particulate was collected on a glass fiber filter (47 mm in diameter) (cat no. APFF04700).

Our previous biomonitoring studies (Culotta et al. 2002, 2005, 2007; Gianguzza et al. 2006a, Gianguzza and Orecchio 2006b; Orecchio and Amorello 2010) indicate that the air of Palermo is seriously polluted, resulting mainly from vehicle exhaust. The high level of total suspended particles (TSP) contain large amount of organic and inorganic micropollutants (PAHs, Pb, Pt, Rh, etc.) (Orecchio and Amorello 2010). In the current work, dust samples for analysis of phthalate esters were collected from thirteen indoor environments (Fig. 1). Indoor dust samples were collected in bedrooms, living rooms, kitchens, corridors, offices, etc. Table 1 gives a descriptive profile of the sampling environments in details.

**Table 1:** Descriptive profile of indoor-sampling sites

No	Indoor location	Characteristic of the location	Location	Ventilation	Age of building
1	Kitchen	Interested by high traffic	City center	Well ventilated	53
2	Studio	Interested by medium traffic	Little suburb	Well ventilated	2
3	Bedroom	Interested by high traffic	City center	Little exposure to sunlight and poorly ventilated	20
3a	Bedroom	Previous station sampled after about eight months	City center	Little exposure to sunlight and poorly ventilated.	20
4	Bedroom	Interested by light traffic	Isolated house	Good ventilation. Well exposed to sunlight	15
5	Bedroom	Apartment fully renovated	Little suburb	Little exposure to sunlight and poorly ventilated	7
6	Kitchen	Very heavy and slow traffic constituted by bus and commercial vehicles		Well ventilated. Well exposed to sunlight	50
7	Bedroom	Distant from traffic, rural area, periodically interested by agricultural practices	Isolated villa	Quite aerated. Well exposed to sunlight	20
8	Living room	Distant from traffic, rural area, periodically interested by agricultural practices	Peripheral villa	Damp and dimly lit. Well ventilated	20
9	Kitchen	Interested by high traffic		Low ventilated	60
10	Bedroom	Interested by very heavy and slow traffic	City center	Little exposure to sunlight and poorly ventilated	93/5^a^
11	Living room	The large road is frequented by light traffic	Industrial/commercial area open area	Well ventilated	23
11a	Bedroom	Previous station	Industrial/commercial area	Well ventilated	23
12	Corridor	The sampling station is located in the Chemical Department	University Campus	Forced recirculation of air	13

^a^Renovated

About 2 g of settled dust samples from each site were collected carefully with brushes from surfaces at a height of 1.5–2.0 m above the ground level, generally at the surface of the furniture so as not to contain foreign coarse material. Hairs and other non-dust particles were removed manually. The samples were refrigerated (4 °C) on site, until they rapidly were transported to the laboratory where they were frozen prior to analysis.

### Chemicals

Analytical-reagent grade dichloromethane, hexane, cyclohexane, and acetone (Riedel-de-Haen, Milano) were used as solvents. A PAEs standard solution containing 6 compounds: dimethyl phthalate (DMP), diethyl phthalate (DEP), di-*n*-butyl phthalate (DnBP), benzyl butyl phthalate (BBZP), bis(2-ethylhexyl) phthalate (DEHP), and di-*n*-octyl phthalate (DnOP) (1,865–1,911 μg/mL) (Mixture EPA Phthalate Esters Mix, Catalog no. 48231) were supplied by Supelco (Milano). Calibration standard solutions with concentrations of 0.5, 1, 5, 10, 20, 30, 40, and 50 mg/L were prepared by diluting the stock standard with solution containing two internal standards. Stock and calibration standard solutions were stored at 4 °C in the refrigerator.

Solution of internal standards (Diethyl phthalate-d_4_ e Bis(2 ethylhexyl) phthalate-d_4_) in hexane was supplied by Supelco, Milano, and used for all analysis. Internal standardization improves precision. They are purposely added to both samples and standards at the same concentration in order to provide a basis for comparison in quantification. Internal standards are especially useful for analyses in which the volume of sample injected to GCMS or the instrument response varies slightly from run to run for reasons that are difficult to control as in case of analyses of traces. Because such errors affect both the internal standard and the analyte peak in the same way, they will tend to cancel out when the ratio of areas is calculated.

Diethyl phthalate-d_4_ was used to quantify DMP, DEP, and DnBP, while bis(2 ethylhexyl) phthalate-d_4_ used for remaining analytes. Solution of di-*n*-hexyl-phthalate-D_4_ (batch SZBA102XV Sigma-Aldrich) as surrogate standard was used in order to determine extraction efficiency.

### Analysis

We carried out different recovery experiments (Soxhlet and ultrasonic bath and different solvent mixtures) by using blank samples dust added of known quantities of PAEs, in order to verify the accuracy and precision of the analytical procedure, being not commercially available for a reference-certified standard of dust containing PAEs. We performed several extraction steps in 24 h on the dust and sample. After the complete PAEs extraction (the absence of PAEs in the blank was confirmed by GC–MS analysis), a known amount of PAEs standard mixture was added to the purified blank sample. The best recoveries 79 ± 7 % were obtained utilizing Soxhlet extraction with cyclohexane.

The detection limit (LOD), estimated as 3 r (three times the background noise) (International Union of Pure and Applied Chemistry (IUPAC) criterion), was similar for all the analyzed compounds (less than 9 μg/kg for all compounds). The blank values of analytical procedure remained always below the quantification limits (LOQ): 30 μg/kg estimated as 10 r (ten times the background noise) (IUPAC criterion).

To evaluate the precision of the method, three replicates of the same dust sample were analyzed. The relative standard deviations of the replicates, on the concentrations of individual compounds, ranged from 0.8 to 10 % and, as reported in a paper by Horwitz and Albert (1997), are satisfactory for the level of concentrations measured in our samples. Before the analysis, for each sample, a known volume (150 μl) of the surrogate standard solution (di-*n*-hexyl-phthalate-D_4_) 100 mg/L was added to determine the yield of the extraction. The recoveries are never less than 79 % and in most cases almost 100 %.

The filter containing atmospheric dust were extracted, in a Soxhlet extractor for 24 h, using cyclohexane and analyzed for PAEs as will be described later for indoor dust. All dust samples (about 100 mg) were extracted in a Soxhlet extractor for 24 h using cyclohexane. The extracts were filtered through a Pasteur pipette filled with anhydrous Na_2_SO_4_, previously rinsed with cyclohexane, and concentrated in a rotary evaporator at *T* = 50 (±0.5) °C. The final volume was around 1 mL. The last stage in the procedure involved drying the PAEs containing solution under a weak nitrogen flow at room temperature. The dry residue was dissolved in 1 mL of solution containing perdeuterated internal standards in cyclohexane.

The separation of the investigated compounds was carried out using a gas chromatograph (Shimadzu mod. GC-17A). The GC instrument was coupled with a mass spectrometer (Shimadzu, quadrupole detector mod. GCMS-QP5000) equipped with an acquisition data system (Shimadzu, CLASS 5000). The injection of both extracts from samples and standard solutions (1 μL) was performed by hand. Any inaccuracy in the measurement of the volumes of the samples and standards injected is minimized by the use of internal standards.

The identification of PAEs in the solutions was carried out on the basis of previously determined retention times and confirmed by using mass spectra. The instrumental data were acquired, initially in scan mode and then in single-ion monitoring mode (SIM). The scan mode allows the identification of all the chemicals contained in the injected solution, while the SIM mode allows quick quantification of compounds using the preselected ion peaks. On the other hand, non-preselected peaks and other pollutants are not quantified.

Quantifications of PAEs in the samples were done with the calibration curves of which the correlation coefficients were all higher than 0.99 relative to the perdeuterated PAEs added to the dry residue. The calibration was repeated every three analysis. The response of the GC–MS instrument was checked every morning using a solution containing only two compounds (DEP and DEHP). The present study demonstrates that the GC–MS in SIM mode analysis, without cleanup step, of PAEs in the settled dust is a suitable method for determination of trace amounts of these compounds.

**Table 2:** Mean concentrations (mg/Kg) phthalates ester in indoor dust in others country

Analita	This paper	Bulgary	Denmark	Germany	Norway	USA	Sweden
DMP	15	260					
DEP	31	170	2	3.1	10	5	
DnBP	799	7,860	15	87	100	20	150
BBzP	99	320	4	24	110	45	135
DEHP	304		210	450	640	340	770
DnOP	41	250					
Total	1,289	176	231	564	860	410	1,055

## Results and discussion

**Fig. 2:**
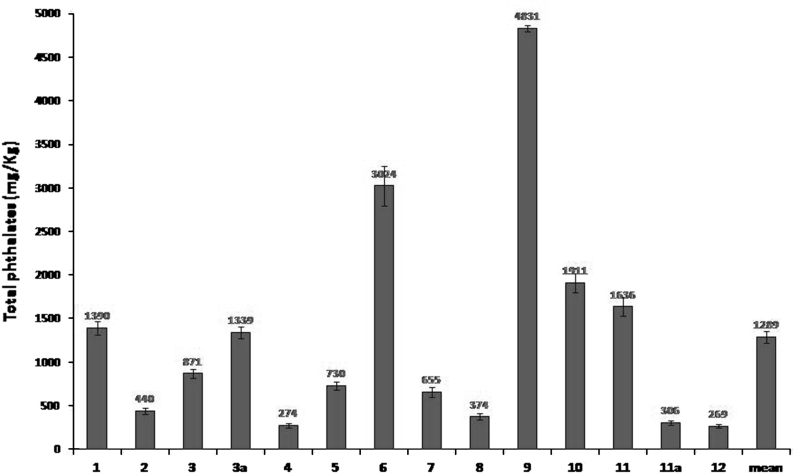
Total and single PAEs (in mg/kg) (average of three analysis) concentrations (corrected for the recovery) in door-settled dust samples

All results (Fig. 2) for PAEs concentrations reported in this paper are given as mean value of triplicate analyses of each sample and are corrected based on recovery. Total PAEs concentrations in outdoor dust samples collected for the present study were less than the quantification limit (about 100 ng/m^3^). These results lead to the conclusion that indoor phthalates are not introduced from outside. Phthalates were found in all investigated indoor stations in Palermo. The total concentrations of PAEs in indoor dusts are presented in Fig. 2 and are in the range from 269 to 4831 mg/kg with a mean of 1,289 mg/kg.

Concentrations of total PAEs determined by us (mean total PAEs 1,289 mg/Kg) are slightly lower than those of other countries (mean total PAEs 2,352 mg/Kg), in particular than those of Bulgaria (total PAEs 8,860 mg/Kg) (Yuan et al. 2002; Langer et al. 2010) (Table 2). This may probably reflect frequent use of PVC flooring in Bulgaria; furthermore, the differences in PAEs concentrations in indoor dust could therefore be due to differences in climate, economy, and lifestyle. For example, in Italy, floors and coverings of PVC are few used probably due to the fact that, in the creation or restructuration of domestic buildings, the owners or builders prefer to use traditional materials of natural origin (tiles ceramic, stoneware, lime, concrete, etc.) (Orecchio 2013). Also, because of direct air exchange between indoor and outdoor, due to open doors and windows all year-round, the concentrations of PAEs in indoor dust in Palermo might be lower than observed in others European areas.

Therefore, we suspect that the results of different researches are hardly comparable, for example, dust samples collected by filter methods contain smaller dust particles than those obtained from vacuum cleaner bags (Bornehag et al. 2005). In our case, we analyze the spontaneously settled dust without any treatment for the selection of particle size because the occupants of confined environments are exposed to this type of material. The surface from which the dust is collected can also influence their resulting chemical constituents. At last, the method of extraction and analysis can also influence the measured concentrations (Bornehag et al. 2005).

The wide range of total PAEs concentrations (mean relative standard deviation = 99 %), found in the settled dust samples taken in account in this paper, indicates that the amount of phthalates greatly varies from site to site and is influenced by several factors, in agreement with a previous research carried out on polycyclic aromatic compounds (Mannino and Orecchio 2008).

The clearest features of the data on total PAEs (Fig. 2) are that isolated or peripheral stations: no. 4 (bedroom of a house built about 15 years ago, having good ventilation), no. 7 (bedroom of an isolated villa), no. 8 (bedroom of an isolated villa quite aerated, built about 20 years ago), and no. 2 (a peripheral studio of a fully renovated apartment with good air circulation, located in a little suburb) have markedly lower total PAEs concentrations than those of environments widely used throughout the year. Generally, in Italy, the isolated houses are used only during summer, holidays, weekends, etc., and so the use of products for the hygiene of the house and personal care and the release from the materials are very limited.

Considering all domestic environments, the highest (mean of results of the same type of environment) total PAEs concentrations were measured in the kitchens (mean concentration = 3,080 mg/kg), while the lower concentrations in the living rooms and studios (mean concentration = 817 mg/kg). In particular, the highest concentrations of PAEs were measured in the stations no. 9 (kitchen of an apartment build around 1950s, low ventilated), no. 6 (kitchen of an apartment build around 1960s), and no. 1(kitchen of an apartment build around 1960s, well ventilated). The high concentrations of PAEs in kitchens, probably depending on the large number of products and materials (plastic goods and kitchen plastic ware), were used in these environments.

Individual PAEs concentrations are shown in Fig. 2. DnBP, found in all the analyzed dust samples, is the predominant (percentage) compound in most of the samples (11/12). In particular, the highest concentrations of DnBP were found in two kitchens: stations no. 6 (2,158 mg/kg = 71 % of total PAEs) and no. 9 (3,127 mg/kg = 65 % of total PAEs). The high concentrations of DnBP observed in these two stations can be attributed to the fact that the compound is utilized in cardboard containers for take-away. These results are in good agreement with the conclusion of a study (Jarosova 2006) that investigated the occurrence of both DnBP and DEHP in food products and packaging materials available on market. Overall, foods contain more DnBP than DEHP. Also, several experiments have been performed within 45 days which is sufficient for the more volatile phthalate (DnBP) to reach equilibrium conditions: DnBP reaches higher concentrations in the air than DEHP (Jarosova 2006). The mass transfer of DEHP in the dust via the gas phase was significantly lower. However, other experiments showed elevated mass transfer of DEHP only in case of direct contact between emission source and sink.

In addition, the presence of DnBP in the above-mentioned stations (no. 6 and no. 9) may be attributed to fact that in the buildings realized after the 1950s were employed, and still exist, several materials that release chemicals into indoor air (Weschler 2009). In these building, for example, flexible PVC insulation replaced rubber and textile braid insulation on wiring and cable.

The highest concentrations of total phthalates are found in poorly ventilated environments and or having little exposure to sunlight. For example, the total PAEs concentration of the stations no. 9 (4,831 mg/kg) and no. 10 (1,911 mg/kg) may be consistent with the fact that slight exposure to sunlight and inadequate ventilation of these environments prolong the lifetime of pollutants attached to the dust by minimizing the natural degradation processes catalyzed by sunlight, while the lowest concentration of total PAEs (269 mg/kg) in the station no. 12 may be justified by the fact that air exchange is constantly ensured by a system of forced recirculation that disperses outside the pollutants generated within the environment and does not allows the accumulation of them in settled dust.

In the sample of dust collected in the Department of Chemistry (no. 12), the percentage distribution of individual compounds is slightly different from that of most stations. The above differences could be attributed to different building materials, use of environments, habits, and their sanitation. In particular, the high percentage (≈50 %) of DEHP, found in the dust sampled in this station (no. 12), is in agreement with the fact that PVC is the flooring material in all common areas (corridors, halls, stairs, bathrooms, etc.) of this building. This type of flooring typically contains 30–40 % of plasticizer (generally DEHP) to remain flexible and avoid brittleness (Bornehag et al. 2005). This plasticizer is emitted over the life of the flooring. Recently, the use of DEHP, as well as BBzP, has been reduced because of concerns regarding potential health effects (Bornehag et al. 2004).

DEP, an environmental ubiquitous chemical (Api 2001), was found at appreciable concentrations only in two little exposed to sunlight and poor ventilated environments: kitchen no. 9 (162 mg/kg) and bedroom no. 5 (134 mg/kg). The very low concentrations, measured in well exposed to light and aerated environments (for example, stations no. 4 and no. 6), suggest that DEP undergoes rapid photo-degradation (Api 2001).The DEP presence in these stations is in agreement with the use of this chemical as an ingredient of detergents formulation, cosmetics, and fragrance preparations at concentrations ranging from 0.1 % to about 30 %. More specifically, diethyl phthalate is used in perfumes and deodorants as fixative and solvent (Sonde et al. 2000) and furthermore in toilet preparations as denaturant for alcohol (Api 2001). In addition, DEP is a component of insecticide sprays and mosquito repellents. More recently, fragrances have been added to certain types of candles (Orecchio 2011) and incense sticks. These products have been utilized since ancient times for religious purposes and are in our day frequently used for different objectives in various indoor environments. Also, DEP is used as a plasticizer for cellulose ester plastic films and sheets in a wide variety of consumer products, including plastic-packaging films, cosmetic formulations (Bonini et al. 2008), and molded and extruded articles such as toothbrushes, tool handles, and toys.

The mean concentration of DEHP in our samples (304 mg/kg) is slightly lower than those of dust sampled in other countries (392 mg/kg); in fact, presence of DEHP in indoor dust is associated with PVC flooring (Bornehag et al. 2005; Clausen et al. 2004). As mentioned before, for domestic buildings, Italians prefer natural materials than synthetic ones (Orecchio 2013).

In our samples, the concentrations of BBzP were lower than DEHP, but the difference may be because BBzP is more strongly linked with PVC than DEHP. DMP was found, at appreciable concentration (188 mg/kg) only in sample no. 9 (kitchen). The other phthalates were detected in all samples. Considering the average of the results of all the stations, DnBP and DEHP, the two most abundant components in settled dust samples, respectively, constitute 62 and 24 % of the total PAEs. These results are in good agreement with a study that showed that more than 90 % of total phthalates present in household waste materials collected from waste management’s were represented by DEHP (Oie et al. 1997).

Considering our analytical results, the percentages of the more volatile PAEs (DMP, DEP) are generally less than those of lower volatile compounds. According to their vapor pressure, we can justify that the analytes having high molecular weight contribute quantitatively to total phthalate esters. In fact, heavier compounds, once produced in the indoor environments, are more likely to be concentrated in dust and are deposited on surfaces, while the more volatile ones are in the gas phase and then more easily disperse. We assume that DMP and DEP found in the some samples are solubilized or adsorbed by organic matter that constitutes the majority of the array.

In one case, we analyzed the settled dust of the same bedroom (no. 3 and no. 3a) of the same house after about 8 months. As can be seen from Fig. 2, concentrations of total phthalates are, respectively, 871 and 1,339 mg/kg, but the most relevant data are that the percentages of the various components are very similar between them. The similarity in the composition makes us assume that the source of phthalate esters is the same environment that depends on the structural characteristics of the house and habits of the occupants. Also, we analyzed the dust of two different rooms (no. 11a living room and no. 11a a bedroom) of the same house. As can be seen from Fig. 2, concentrations of total phthalates differ greatly (1,326 and 306 mg/kg), but the most relevant data are that the percentages of the various components are different between them. These data makes us assume that the source of phthalate esters is the different environments that depend on the utilized products and lifestyle of the occupants.

A linear correlation between total PAEs concentration and a single compound content, with the exclusion of the two most volatile components, was calculated. The values of r for the compounds were ranged from 0.69 to 0.96. The results suggest that most of the PAEs identified in the samples of settled dust originate from the same type of material. This evidence indicates that during the use of an indoor environment, a characteristic mixture of PAEs was produced.

In this study, principal component analysis (PCA) was applied to evaluate the similarities and differences of distribution patterns for single PAEs in indoor-settled dust. The statistically significant correlation among DEP, DnBP, BBzP, and DMP (Pearson correlation coefficient from 0.660 to 0.759) confirms that some PAEs have common sources and are with similar environmental behavior.

We also note differences in phthalate concentrations between buildings from different construction periods; the total PAEs concentration was higher in ancient homes compared to those constructed later. A linear correlation between total PAEs concentration and age of the building was calculated (*R* = 0.71). The value of r increases if we consider single BBZP (*R* = 0.84) and DEHP (*R* = 0.92). Given the uncertainty regarding the age of buildings and the limited number of experimental data, statistical considerations have not been affected. From our data, we can assume that, the people who live in old houses are more exposed than those who live in latest buildings, and this is in agreement with the previous literature (Bornehag et al. 2005). This shows that a trend to reduce or remove certain hazardous compounds from building materials and consumer goods has started several years ago and has led to a certain decrease in volatile contaminants emissions.

Sources of phthalates in considered stations are not obvious. In private houses considered in this paper, we did not find PVC as coating or floor. We can assume that polishing products and old building materials could be a predominant source for phthalates.

## Conclusions

There presented method includes advantages of high sensitivity, high selectivity, and low costs. Differently from the classical methods for the evaluation of the quality of indoor air, settled dust can be employed as suitable passive samplers for airborne PAEs pollutants. This method is not substitutive of classical methods but offers an additional source of information. In such away, the need for long periods of sampling with complex, difficult to handle instruments and numerous analytical calculations, is avoided. Moreover, the results obtained with the analysis carried out on a sample of settled dust provide a mean information from the time of the last removing of the dust until final collection, uninfluenced, as occured with classical analysis, by instantaneous conditions at the time of sampling. Also, stored samples of settled dust can be used for retrospective contaminant analysis, if the set of samples extends over long periods and they have been collected and stored in the same manner over time, this approach can potentially provide a reliable date record of changes in the analyte concentration of this media. Owing to the fact that this type of survey is more rapid and inexpensive, it may be used for private citizens who wish to get to know the environment in which they live.

It is very difficult to apportion the contribution of single source to the total pollutant measured in each case because there are a number of other unknown sources for phthalates indoors. However, the number of chemicals present in building materials is still increasing (Weschler 2009); in fact, in recent years, industries are developing and experimenting new substances (adipates, acetates, etc.) that can replace phthalates, primarily DEHP. In light of this, it is necessary to continue monitoring new classes of compounds which are often not well-known toxicological characteristics.
